# Interferon-γ-Directed Inhibition of a Novel High-Pathogenic Phlebovirus and Viral Antagonism of the Antiviral Signaling by Targeting STAT1

**DOI:** 10.3389/fimmu.2019.01182

**Published:** 2019-05-28

**Authors:** Yun-Jia Ning, Qiong Mo, Kuan Feng, Yuan-Qin Min, Mingyue Li, Dianhai Hou, Cheng Peng, Xin Zheng, Fei Deng, Zhihong Hu, Hualin Wang

**Affiliations:** ^1^State Key Laboratory of Virology, Wuhan Institute of Virology, Chinese Academy of Sciences, Wuhan, China; ^2^University of Chinese Academy of Sciences, Beijing, China; ^3^Department of Infectious Diseases, Union Hospital, Institute of Infection and Immunology, Tongji Medical College, Huazhong University of Science and Technology, Wuhan, China

**Keywords:** IFN-γ, antiviral immunity, STAT1, severe fever with thrombocytopenia syndrome virus (SFTSV), NSs, inclusion body, immune evasion, virus-host interaction

## Abstract

Severe fever with thrombocytopenia syndrome (SFTS) is a life-threatening infectious disease caused by a novel phlebovirus, SFTS virus (SFTSV). Currently, there is no vaccine or antiviral available and the viral pathogenesis remains largely unknown. In this study, we demonstrated that SFTSV infection results in substantial production of serum interferon-γ (IFN-γ) in patients and then that IFN-γ in turn exhibits a robust anti-SFTSV activity in cultured cells, indicating the potential role of IFN-γ in anti-SFTSV immune responses. However, the IFN-γ anti-SFTSV efficacy was compromised once viral infection had been established. Consistently, we found that viral nonstructural protein (NSs) expression counteracts IFN-γ signaling. By protein interaction analyses combined with mass spectrometry, we identified the transcription factor of IFN-γ signaling pathway, STAT1, as the cellular target of SFTSV for IFN-γ antagonism. Mechanistically, SFTSV blocks IFN-γ-triggered STAT1 action through (1) NSs-STAT1 interaction-mediated sequestration of STAT1 into viral inclusion bodies and (2) viral infection-induced downregulation of STAT1 protein level. Finally, the efficacy of IFN-γ as an anti-SFTSV drug *in vivo* was evaluated in a mouse infection model: IFN-γ pretreatment but not posttreatment conferred significant protection to mice against lethal SFTSV infection, confirming IFN-γ's anti-SFTSV effect and viral antagonism against IFN-γ after the infection establishment. These findings present a picture of virus-host arm race and may promote not only the understanding of virus-host interactions and viral pathogenesis but also the development of antiviral therapeutics.

## Introduction

Severe fever with thrombocytopenia syndrome (SFTS) is an emerging hemorrhagic fever-like disease caused by SFTS virus (SFTSV), a member of the *Phlebovirus* genus, *Phenuiviridae* family, *Bunyavirales* order (1–3). SFTS was listed as one of the top priority diseases for research and development by World Health Organization due to its high case-fatality rate between 12 and 30%, the lack of specific medical countermeasures, the multiple transmission routes (including tike bites, human-to-human contacts, and potential aerosol dissemination), and the trend of wider distribution (4, 5). SFTSV was indentified firstly in China and subsequently also recognized in South Korea (6, 7) and Japan (8). Following its identification, SFTSV has become a representative species of phleboviruses as a notable life-threatening pathogen attracting great research interests internationally.

As with the other phleboviruses, SFTSV contains a three-segmented negative-stranded RNA genome (1). The Large (L) segment encodes the RNA-dependent RNA polymerase (RdRp); the medium (M) segment encodes the envelope glycoprotein (GP); and the small (S) segment encodes the nucleoprotein (NP) and a nonstructural protein (NSs) in an ambisense orientation (1, 9). Although the viral pathogenesis is largely unclear, previous studies by us and others have suggested that SFTSV NSs induces cytoplasmic inclusion body (IB) formation and suppresses host type I and III interferon (IFN) responses by sequestering some important antiviral signaling components into the viral IBs (10–13). The antagonism of type I and III IFN system by the NSs- and IB-associated mechanisms could profoundly contribute to the efficient viral replication and high pathogenicity (14–18).

IFNs are secreted cytokines that are grouped into three types: I, II, and III (19, 20). These cytokines play a variety of roles in innate and adaptive immunity, especially by triggering the Janus kinase (JAK)-signal transducer and activator of transcription (STAT) signaling transduction (19–22). Among the three IFN types, there are multiple subtypes for type I IFNs (such as IFN-β and various IFN-α) and type III IFNs (including IFN-λ1, IFN-λ2, IFN-λ3, and IFN-λ4) (20), while there is only a single type II IFN, IFN-γ (23). Moreover, type I and III IFNs also share other significant commonalities, in comparison to IFN-γ. For instance, type I and III IFNs engage the common STAT transcription complex (containing both STAT2 and STAT1 which further assemble with IFN regulatory factor 9 to form a heterotrimer) to mount host antiviral responses (20), while IFN-γ directs a different JAK-STAT signaling that involves a STAT1 homodimer as the downstream transcription regulator (20, 23). In function, type I and III IFNs are the primary antiviral IFNs which exhibit strong and general antiviral activities; however, IFN-γ appears to be important for immunity *in vivo* to only a small number of viruses, but together with various bacteria, fungi, and parasites (20, 24–26).

During SFTSV infection, the antiviral type I and III IFNs likewise play significant roles in restriction of the viral infectivity and pathogenicity (14–16) and moreover in turn are antagonized by the virus (10–13, 27). However, whether the type II IFN, IFN-γ, has anti-SFTSV activity and whether it can be counteracted are undetermined. Here, we demonstrate both a clear IFN-γ-directed inhibition of SFTSV infection *in vitro* and *in vivo* and the viral counteraction against IFN-γ antiviral signaling by function and mechanism studies, highlighting a notable evolutionary arm race between the virus and host. The study sheds light on the viral pathogenesis and virus-host interactions and may benefit the future development of antiviral therapies.

## Materials and Methods

### Clinical Serum Analysis

During the period April 2016 to July 2017, acute phase serum samples were collected from 33 SFTS patients who were admitted to the Union Hospital of Tongji Medical College, Huazhong University of Science and Technology (Wuhan, China). In addition, serum samples from 17 healthy volunteers were correspondingly included in this study as controls. The concentrations of serum IFN-γ were analyzed by enzyme-linked immunosorbent assay (ELISA) using commercial kits (DAKEW Biotech, China) in accordance with the manufacturer's instructions. The study was conducted basically as described previously (28–30) and approved by both the ethics committees of Wuhan Institute of Virology, Chinese Academy of Sciences and the Union Hospital of Tongji Medical College, Huazhong University of Science and Technology.

### Cell and Virus

HepG2, HEK293, and Vero cells were cultured in Dulbecco's modified Eagle's medium (DMEM; Gibco) supplemented with 10% new-born calf serum at 37 °C in a 5% CO2 atmosphere. HEK293T cells were cultured in DMEM containing 10% fetal bovine serum (FBS; Gibco). SFTSV was expanded in Vero or HEK293 cells and manipulated in a biosafety level-3 laboratory as previously described (10, 13, 18). Virus titers were determined by 50% tissue culture infectious dose (TCID50) method (18).

### Plasmid, Antibody, and Reagent

The firefly luciferase reporter plasmid for IFN-γ-responsive promoter and the *Renilla* luciferase control plasmid (pRL-TK) (31–33) were kindly provided by Dr. Hong-Bing Shu (Wuhan University, China). The NSs or NP expression plasmids were constructed as described previously (10, 13, 18). Mouse anti-NSs antiserum or rabbit antisera to NSs, NP, GP (N-terminal domain), or RdRp were respectively raised against the corresponding viral proteins generated by *Escherichia coli* (10, 18). Rabbit antibodies to S-tag (Abcam), STAT1 (Cell Signaling Technology), or STAT2 (Santa Cruz Biotech) and mouse antibodies to HA-tag (Beyotime), β-actin (Beyotime), or STAT1 (Santa Cruz Biotech) were purchased from the indicated manufacturers. Secondary antibodies include goat anti-mouse IgG-fluorescein isothiocyanate (FITC) (Proteintech), goat anti-rabbit IgG-Rhodamine (Chemicon), and goat anti-rabbit IgG-Cy5 (Abcam). Recombinant mouse or human IFN-γ proteins were purchased from Cell Sciences or Peprotech Inc., respectively. Recombinant human IFN-α was from PBL Biomedical Laboratories.

### Reporter Gene Assay

HEK293 cells maintained in 24-well plates were cotransfected with 50 ng of the IFN-γ reporter plasmid and 10 ng of the control plasmid pRL-TK, together with 600 ng of the NSs expression plasmid or empty vector control using lipofectamine 3000 (Invitrogen) according to the manufacturer's instructions. Twenty-four hours post transfection, cells were treated with human IFN-γ (50 ng/ml) or left untreated for 16 h, followed by the measurement of luciferase activities with a dual-luciferase reporter assay kit (Promega). For data presentation, the firefly luciferase activity was normalized to *Renilla* luciferase activity to show the relative enzyme activity and furthermore fold activation over the untreated groups was also calculated.

### Real-Time Quantitative PCR

The mRNA levels of host genes were analyzed by real-time PCR as previously described (13, 18). Briefly, total RNA were purified from HepG2 or HEK293 cells using TRIzol reagent (Life Technologies). Reverse transcription then was performed to obtain cDNA with a reverse transcription kit from Promega. Real-time quantitative PCR was conducted using an SYBR Green real-time PCR kit (Toyobo) with the corresponding gene primers listed in Table 1. Relative mRNA levels were calculated by the 2-ΔΔCT method with the *GAPDH* mRNA as internal control.

**Table 1:** List of primers for real-time quantitative PCR.

**Primer name*^***a***^***	**Primer sequence (5′ to 3′)**
OAS2-F	CGGTGTATGCCTGGGAACAGG
OAS2-R	GGGTCAACTGGATCCAAGATTAC
IP-10-F	GCCTCTCCCATCACTTCCCTAC
IP-10-R	GAAGCAGGGTCAGAACATCCAC
STAT1-F	ATCAGGCTCAGTCGGGGAAT
STAT1-R	TGGTCTCGTGTTCTCTGTTCTGC
STAT2-F	TATCTCTTGCCCTTCCTACTCCTC
STAT2-R	ATGTTATGCTTTCACCTCTCACCC
BCL-F	ACACCCCCTTTCTGCTGACAT
BCL-R	GCGGTGTCTGCCGTAGGTT
GAPDH-F	ACCACAGTCCATGCCATCAC
GAPDH-R	TCCACCACCCTGTTGCTGTA

a *F, the forward primer; R, the reverse primer*.

### Immunofluorescence and Confocal Microscopy

Immunofluorescence assays combined with confocal microscopy were used to monitor protein expression and subcellular localization as previously described (10, 34). Briefly, following fixation with 4% paraformaldehyde in PBS, transfected or infected cells were permeabilized by 0.5% Triton X-100 in PBS. After blocking with 2.5% bovine serum albumin (Biosharp) and 2.5% normal goat serum (Jackson ImmunoResearch) in PBS, cells were incubated with the primary antibodies overnight at 4°C and then stained with the corresponding secondary antibodies for 1 h at room temperature. Nuclei were visualized by staining with Hoechst 33258 (Beyotime). Images were obtained and analyzed using a Nikon confocal microscope together with the Volocity (PerkinElmer Life Sciences) and Image-Pro Plus (Media Cybernetics, Inc) software.

### Protein Interaction, LC-MS/MS, and Western Blot Analysis

Protein interactions were analyzed by the S-tag pulldown assays using S-protein agarose beads (Merck Novagen) as previously described (10, 13, 18). The coprecipitates were then subjected to mass spectrometry or Western blot (WB) analyses. For mass spectrometric analysis, purified NSs-associated proteins or agarose bead-binding products (as control) were respectively subjected to ingel digestion with trypsin, following sodium dodecyl sulfate–polyacrylamide gel electrophoresis (SDS-PAGE) (35). Then, the tryptic peptides were analyzed by liquid chromatography-tandem mass spectrometry (LC-MS/MS) using a nano-LC-equipped TripleTOF 5600 system (AB SCIEX). The raw tandem spectra were searched against Unified Protein Database (UniProt) with ProteinPilot Software 5.0 (AB SCIEX). The obtained data were based on a false discovery rate (FDR) ≤1% confidence for protein identification. Tandem spectra of representative peptides (identified with >99% confidence) were selectively shown. For WB analysis, protein samples were resolved by SDS-PAGE, followed by the transfer onto polyvinylidene difluoride (PVDF) membranes (Millipore). The membranes were then blocked with 5% BSA in Tris-buffered saline-Tween 20 (TBS-T) and probed successively with primary antibodies and the corresponding horseradish peroxidase-conjugated secondary antibodies (Proteintech). An enhanced chemiluminescence kit (Thermo Fisher Scientific) was used for protein band detection. In some results, protein band intensity was further analyzed with ImageJ software (National Institutes of Health, USA) as indicated.

### Animal Experiment

Three-day-old ICR mice (Beijing Vital River Laboratory Animal Technology Co., Ltd.) were employed for the animal infection experiments. In the IFN-γ pretreatment experiments, indicated doses of mouse IFN-γ (0.05 or 0.5 μg per animal) or medium solvent control were administered by intraperitoneal injection to the suckling mice (*n* ≥ 9/treatment) 24 h prior to infection of SFTSV (1.5×10^3^ TCID50) by intracerebral inoculation. Control mice were administered culture medium instead of virus and kept in separate cages from the infected groups. Survival rates and body weights were monitored and recorded at indicated times following virus challenge. For the IFN-γ treatments post infection, IFN-γ was administered 24 h following SFTSV infection (*n* ≥ 8/treatment). The animal experiments were approved by the Institutional Animal Ethical Committee of Wuhan Institute of Virology, Chinese Academy of Sciences and conducted under the guidelines of the Care and Use of Laboratory Animals (the Ministry of Science and Technology, China).

### Statistical Analysis

Statistical analyses were performed with GraphPad Prism (Version 6) and IBM SPSS (Version 19) software. In the animal experiments, Mantel-Cox log-rank tests and Gehan-Breslow-Wilcoxon tests were used to analyze differences in survival. Two-tailed Student's *t* tests or Mann-Whitney *U* tests were used for comparing mean values in the other experiments of this study. *P*-values < 0.05 were considered statistically significant.

## Results

### Levels of IFN-γ in SFTS Patients and Healthy Individuals

**Figure 1:**
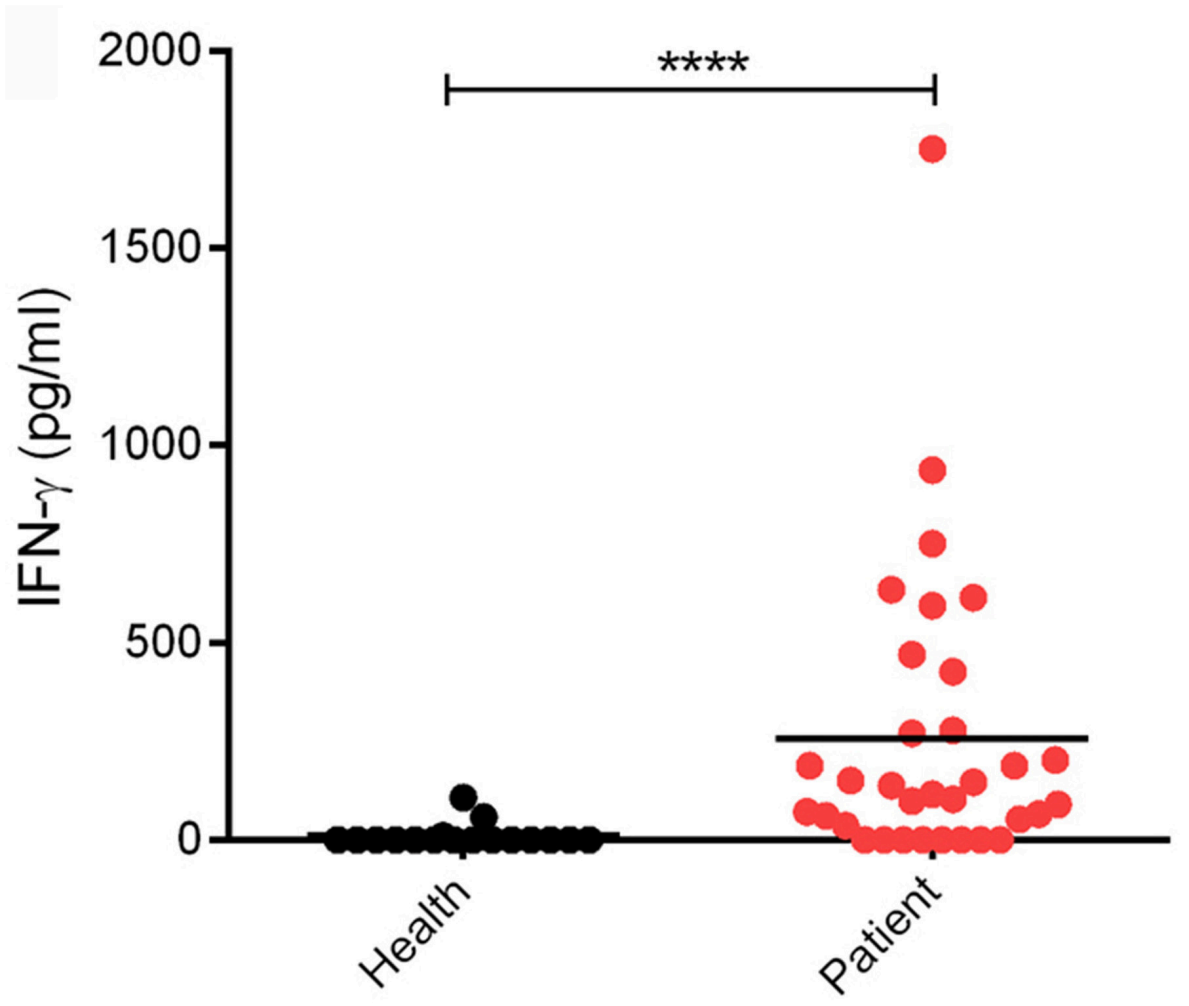
Levels of IFN-γ in SFTS patients and healthy individuals. IFN-γ in serum samples from SFTS cases (*n* = 33) and healthy donors (*n* = 17) was detected by ELISA. Each dot represents the IFN concentration in an individual. Horizon bars indicate the respective group mean. ****P < 0.0001.

Clinically, SFTSV infection can cause a cytokine storm accompany with abnormal expression of many cytokines, whereas whether SFTSV infection induces substantial production of IFNs is largely controversial (16, 36–42). In order to investigate the interplays between SFTSV infection and IFN-γ signaling, we detected the expression level of IFN-γ in SFTS patient sera collected in Hubei Province of China during 2016–2017. In our investigation, the IFN-γ level in SFTS patients was significantly higher than that in the healthy control (Figure 1), revealing remarkable IFN-γ induction responses upon SFTSV infection.

### IFN-γ Suppresses SFTSV Infection in Cell-Based Assays

**Figure 2:**
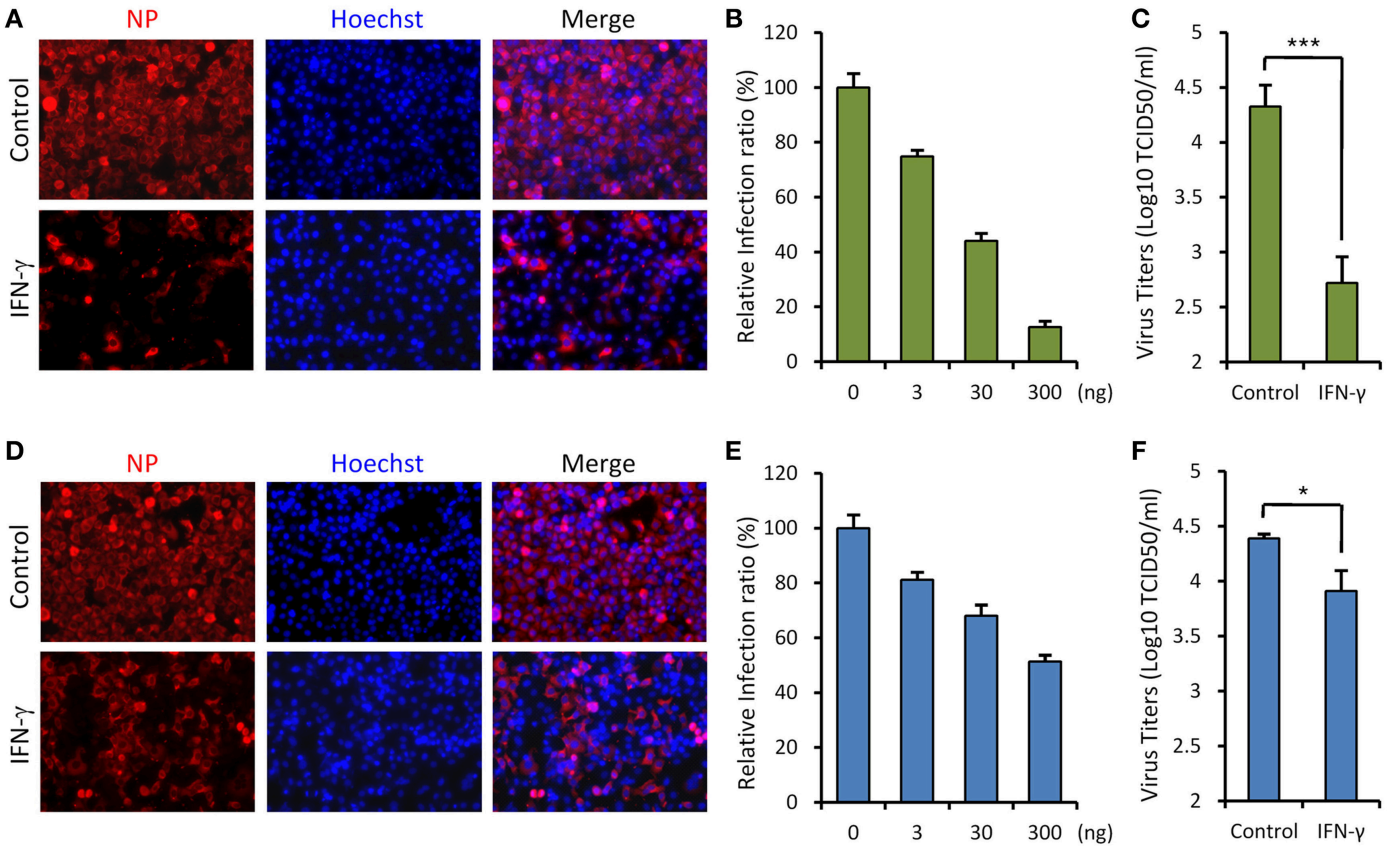
IFN-γ suppresses SFTSV infection *in vitro*. **(A)** HepG2 cells were treated with IFN-γ (200 ng/ml) for 6 h prior to the infection with SFTSV (MOI = 3). Twenty-four hours post infection (p.i.), cells were fixed for immunofluorescence assay (IFA) using the antibody against NP. Nuclei were stained with Hoechst 33258 as shown in blue. **(B)** Cells were pretreated with the indicated doses of IFN- γ and then infected with SFTSV (MOI = 0.1). At 24 h p.i., cells were fixed for IFA as in **(A)**. Percentages of infected cells in the IFN-γ-treated groups were normalized to the infection percentage of the untreated group. **(C)** Cells were pretreated with IFN-γ prior to the infection of SFTSV. At 24 h p.i., virus titers in the culture media were measured by the TCID_50_ method. **(D)** Cells were infected with SFTSV and then treated with IFN-γ at 4 h p.i. Twenty-four hours post infection, cells were fixed and treated as in **(A)**. **(E)** Cells were treated with the indicated doses of IFN-γ at 4 h following SFTSV infection. Relative infection ratios were calculated as in **(B)**. **(F)** Cells were treated with IFN-γ at 4 h following SFTSV infection. At 24 h p.i., virus titers in the culture media were measured as in **(C)**. Graphs show means ± standard deviation (SD), *n* = 3. *P < 0.05; ***P < 0.001.

Since SFTSV infection clinically induces expression of IFN-γ, we asked whether IFN-γ can directly trigger cellular anti-SFTSV defense. To test the antiviral activity of IFN-γ against SFTSV *in vitro*, we treated HepG2 cells with IFN-γ prior to or after SFTSV infection and then examined viral protein expression in cells and infectious virion production in culture media, respectively. As shown in the immunofluorescence assays (IFA), IFN-γ pretreatment evidently inhibits viral NP expression in a dose-dependent manner (Figures 2A,B). Consistently, the virus titer in culture media was greatly decreased by IFN-γ pretreatment (Figure 2C), indicating the robust antiviral activity of IFN-γ against SFTSV when used prior to viral infection. On the other hand, when IFN-γ was applied 4 h post infection (p.i.), SFTSV protein expression and replication were also suppressed (Figures 2D–F); however, the inhibitory effects appeared to be weaker than those exhibited in the IFN-γ-pretreatment assays. These results suggest that IFN-γ has significant anti-SFTSV activities and can inhibit viral protein expression and replication, whereas SFTSV seems to be conferred some resistance to IFN-γ treatment once the viral infection has been established.

### SFTSV NSs Antagonizes IFN-γ Signaling

**Figure 3:**
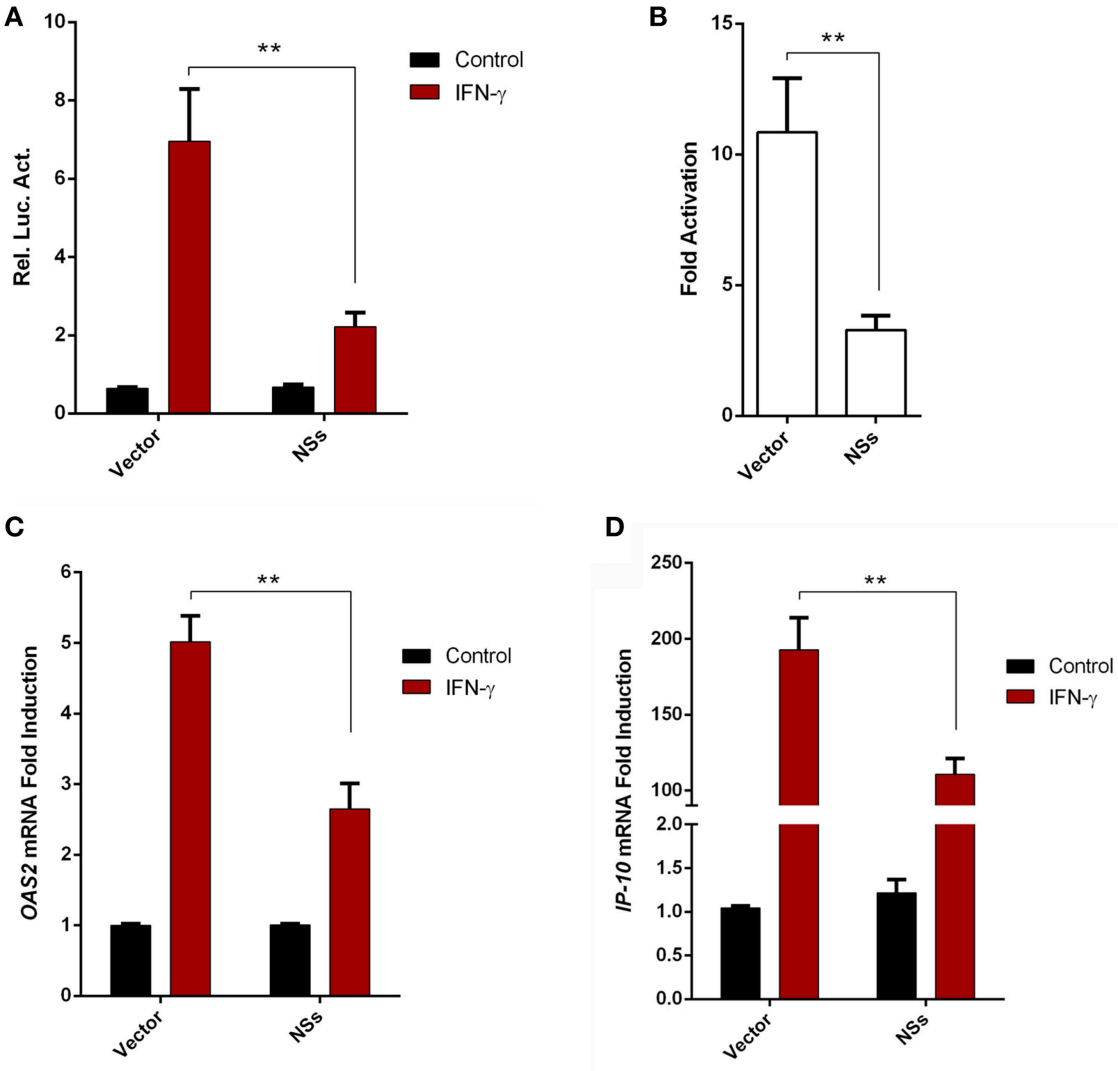
SFTSV NSs antagonizes IFN-γ signaling. **(A,B)** HEK293 cells were cotransfected with the reporter plasmid of IFN-γ-responsive promoter and the *Renilla* luciferase control plasmid (pRL-TK), along with an empty control plasmid (vector) or the NSs expression plasmid. Twenty-four hours after transfection, cells were treated with IFN-γ or left untreated for 16 h, followed by the measurement of luciferase activities. Relative luciferase activity (Rel. Luc. Act.) **(A)** and the fold activation (over the untreated controls) **(B)** were presented, respectively. **(C,D)** HepG2 cells were transfected with the NSs expression plasmid or the vector and at 24 h post transfection, cells were treated with IFN-γ for 10 h or left untreated, followed by the analyses of *OAS2* and *IP-10* mRNA expression with real-time quantitative PCR. Graphs show means ± SD, *n* = 3. **P < 0.01.

Given the resistance of SFTSV to IFN-γ treatment after the viral infection establishment, we considered that viral infection or protein expression may lead to viral counteraction against IFN-γ signaling. As the NSs proteins of bunyaviruses including SFTSV seems to have multiple functions in interference of cellular biological processes (9, 10, 13, 17, 43), we next analyzed the potential of NSs to affect IFN-γ signaling. As shown in the reporter gene assays, activation of IFN-γ-responsive promoter by IFN-γ was significantly repressed in the presence of NSs (Figures 3A,B). Furthermore, NSs expression also inhibited IFN-γ-triggered mRNA expression of interferon-stimulated genes (ISGs), *2*′*-5*′*-oligoadenylate synthetase 2* (*OAS2*) and *IFN*-γ*-inducible protein 10* (*IP-10*) (Figures 3C,D). These results demonstrate that NSs is an antagonist of IFN-γ signaling, likely contributing to the resistance of IFN-γ by SFTSV.

### Identification of STAT1 as the Cellular Target of SFTSV NSs for IFN-γ Signaling Suppression

**Figure 4:**
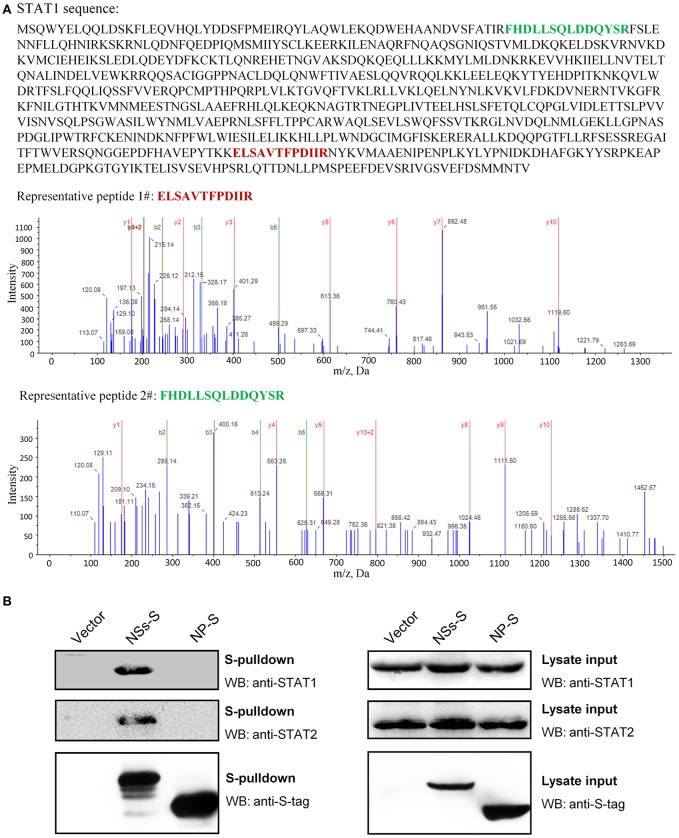
Identification of STAT1 as the NSs target for IFN-γ signaling suppression. **(A)** Results of mass spectrum analysis. The purified NSs-associated proteins or the agarose bead-binding products (control) were subjected to LC-MS/MS analysis. STAT1, the key transcription factor in IFN-γ signaling pathway, was specifically identified in the NSs coprecipitates. The tandem spectra of two representative peptides (identified with >99% confidence) of STAT1 (accession, NP_009330) were shown. **(B)** Validation of the NSs-STAT1 interaction. HEK293 cells were transfected with the control plasmid (vector) or plasmids encoding NSs-S or NP-S. At 24 h post-transfection, protein interactions were examined by pulldown assay. Subsequently, pulldown products and cell lysates (lysate input) were subjected to WB analyses using the indicated antibodies.

To unravel the molecular mechanism underlying NSs-mediated inhibition of IFN-γ signaling, we firstly identified the potential cellular target(s) of NSs by S-tag protein pulldown (S-pulldown) combined with LC-MS/MS analysis. HEK293T cells transfected with the control plasmid or the plasmid expressing NSs C-terminally fused with an S-tag (NSs-S) were lysed for S-pulldown assay at 24 h post transfection using the S-protein agarose beads (10, 13, 18). The pulldown products were then subjected to trypsin digestion and LC-MS/MS analysis. Interestingly, the key transcription factor STAT1 (but not other host molecules) of the IFN-γ signaling pathway was identified specifically in the NSs co-precipitates (Figure 4A). In total, eight STAT1 peptides were recognized with high confidence (data not shown) and the tandem spectra of two representative peptides (identified with ≥99% confidence) were shown in Figure 4A.

Furthermore, the interaction of NSs with STAT1 was validated by pulldown and WB analysis. As shown in Figure 4B, endogenous STAT1 as well as the main NSs target of type I and III IFN signaling cascades, STAT2 (previously identified and included here as control), was indeed detected specifically in the pulldown products of NSs but not NP or the vector group. Together, these data indicate that NSs interacts with STAT1 which is likely the target of NSs for IFN-γ signaling antagonism.

### NSs Suppresses IFN-γ-Induced Nuclear Translocation of STAT1 by Arresting STAT1 Into IBs

**Figure 5:**
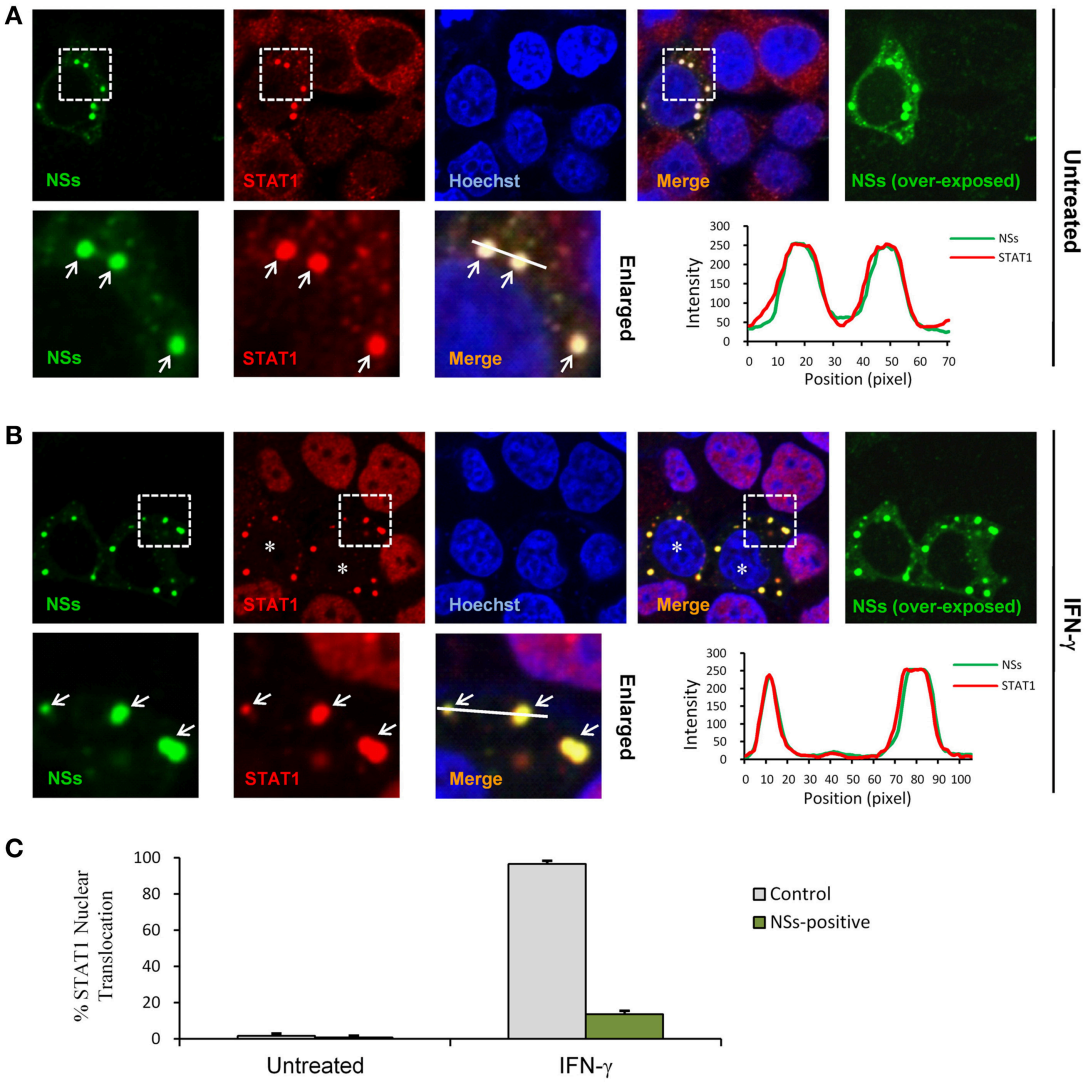
NSs blocks IFN-γ-induced nuclear translocation of STAT1 by trapping STAT1 into IBs. **(A,B)** HEK293 cells transfected with the NSs expression plasmid were left untreated or treated with IFN-γ (as indicated) for 30 min at 48 h posttransfection and then fixed to visualize the expression and localization of NSs (green) and endogenous STAT1 (red) by IFA and confocal microscopy. Nuclei stained with Hoechst were shown in blue. Asterisks indicate the blockade of STAT1 nuclear accumulation in NSs-expressing cells. Arrows in the enlarged images of the dotted box areas show the sequestration of STAT1 into NSs IBs. An intensity line graph in the lower right corner of each panel shows the signal intensity of the green and red channels along the line in the enlarged merged images. To better visualize the NSs-positive cells, over-exposed images of the green/NSs channel were also presented. **(C)** Cells with or without NSs expression from the experiments of **(A,B)** were respectively scored for STAT1 nuclear translocation. Approximately 100 cells were counted for each group. Percentages of cells with noticeable STAT1 nuclear accumulation were shown, respectively.

STAT1 is the key component downstream of IFN-γ signaling which is activated and translocalized from cytoplasm into nucleus upon IFN-γ stimulation to mount antiviral and immunoregulatory gene expression (23). In contrast, SFTSV NSs is located in cytoplasmic IBs induced by NSs itself (10). Considering the NSs-STAT1 interaction, we investigated whether NSs expression affects the nuclear translocation of STAT1 in response to IFN-γ treatment. HEK293 cells were transfected with the NSs expression plasmid and treated with IFN-γ or left untreated for 30 min, followed by fixation and IFA. As shown in Figures 5A,B, STAT1 was efficiently arrested into NSs IBs, establishing a clear NSs-STAT1 colocalization irrespective of IFN-γ treatment. Furthermore, IFN-γ treatment induced STAT1 accumulation into nuclei, whereas the nuclear accumulation was dramatically blocked in NSs-expressing cells where STAT1 was still sequestered in NSs IBs (Figures 5B,C). These observations suggest that NSs can impede IFN-γ-elicited STAT1 signaling by hijacking STAT1 into IBs and hence blocking the nuclear translocation of the transcription factor.

### In the Context of Viral Infection, SFTSV Antagonizes IFN-STAT1 Signaling via Not Only the Sequestration of STAT1 Into NSs IBs but Also a Potential Down-Regulation of STAT1 Abundance

**Figure 6:**
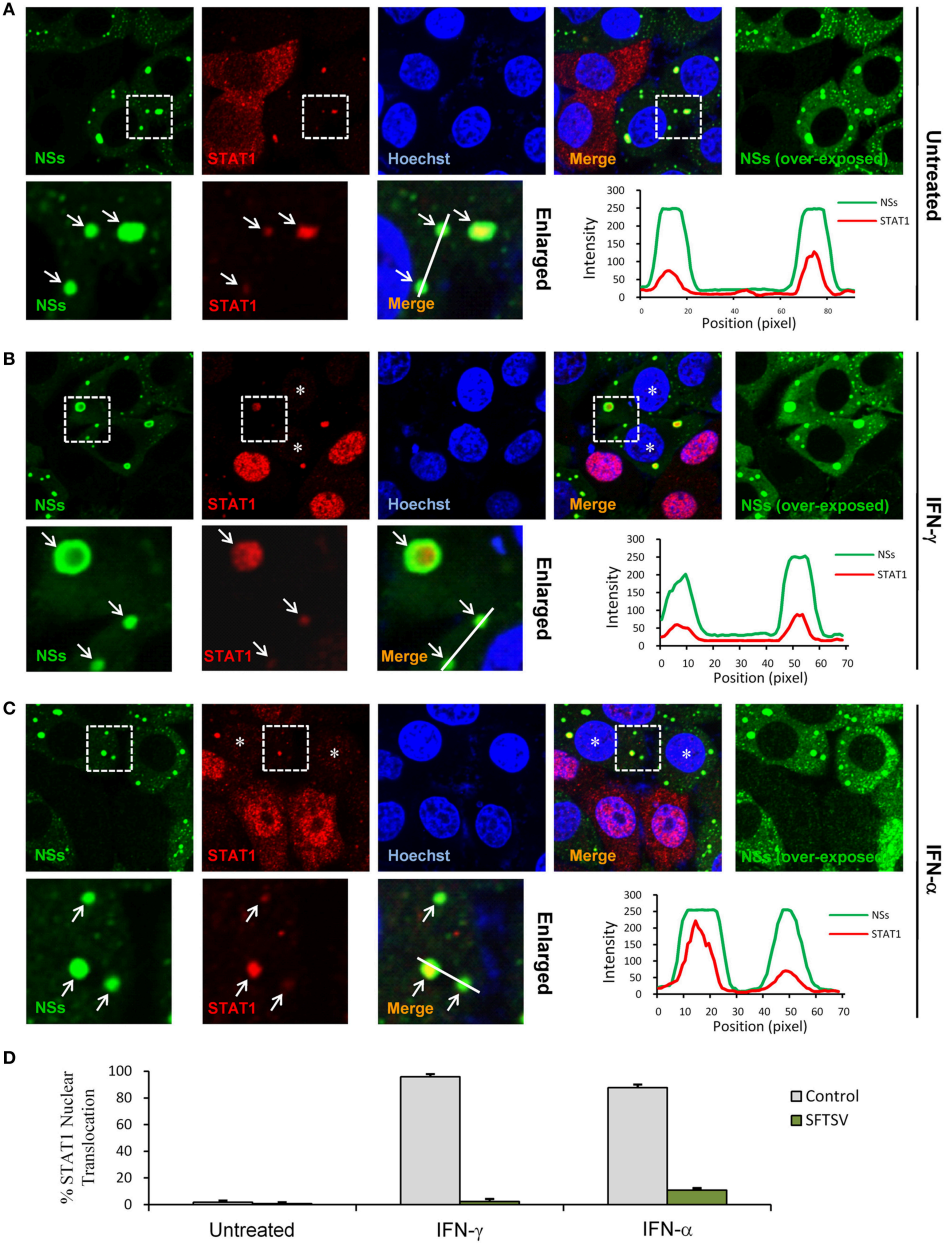
SFTSV antagonizes IFN-STAT1 signaling via sequestration of STAT1 into NSs IBs and down-regulation of STAT1 abundance. **(A–C)** HEK293 cells infected with SFTSV were left untreated **(A)** or treated with IFN-γ **(B)** or IFN-α **(C)** for 30 min at 48 hpi and fixed for visualizing the expression and localization of NSs (green) and endogenous STAT1 (red) by IFA. Nuclei were stained with Hoechst. Asterisks indicate the deprivation of STAT1 nuclear accumulation in SFTSV-infected cells. Arrows in the enlarged panels show the sequestration of STAT1 into SFTSV NSs IBs. The intensity line graphs show the green and red signal intensity along the white lines in enlarged merged images. Over-exposed images of the green/NSs channel were also presented to visualize all the infected cells. **(D)** Cells with or without NSs expression from the experiments of **(A–C)** were respectively scored for STAT1 nuclear translocation. Percentages of cells with noticeable STAT1 nuclear import were shown.

We next investigated the effects of NSs expression on STAT1 subcellular location and translocation in response to IFN-γ in the context of viral infection. Indeed, the trapping of STAT1 by NSs IBs could be observed in SFTSV-infected cells as well (Figure 6A), consistent with the condition of NSs transient expression (Figure 5A) and our previous observations (13). However, unexpectedly, we also noticed that the overall immunofluorescence signals of STAT1 in SFTSV-infected cells appeared to be weaker compared to those in the uninfected cells (Figures 6A–C) or NSs transient expression cells (Figures 5A,B), indicating that SFTSV infection may be able to cause a down-regulation of STAT1 abundance which cannot be achieved by NSs expression alone. Furthermore, both signal reduction and NSs IB hijacking of STAT1 were still noticeable in SFTSV-infected cells upon IFN-γ treatment, leading to a remarkable deprivation of STAT1 nuclear accumulation (Figures 6B,D). STAT1 is also a crucial component of type I IFN signaling. Thus, with the present experimental settings, we then analyzed the impact of SFTSV infection on IFN-α-triggered STAT1 nuclear translocation. Interestingly, after IFN-α treatment, the two effects of SFTSV infection on STAT1 (sequestration in NSs IBs and abundance reduction) were found in SFTSV-infected cells as well and consequently STAT1 nuclear accumulation was similarly diminished (Figures 6C,D). Collectively, these data demonstrate that SFTSV likely blocks IFN-STAT1 signaling by two mechanisms, i.e., NSs sequestration of STAT1 in IBs and viral infection-caused decrease of STAT1 abundance.

### Down-Regulation of STAT1 Abundance at the Protein Level by SFTSV Infection but Not Expression of NSs or the Other Viral Proteins

**Figure 7:**
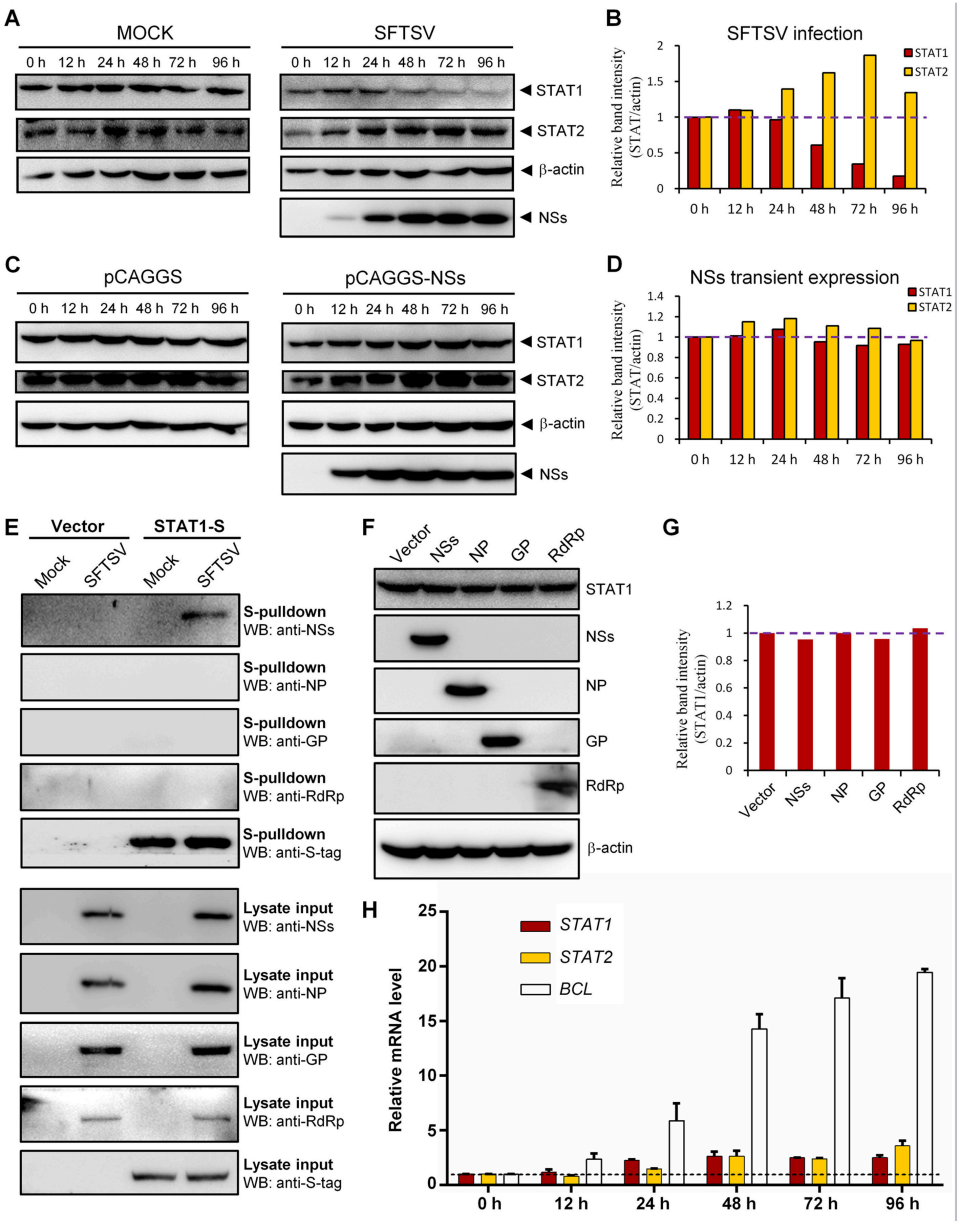
Down-regulation of STAT1 abundance at the protein level by SFTSV infection but not expression of NSs or the other viral proteins. **(A–D)** HEK293 cells were mock infected or infected with SFTSV **(A)**, or transfected with the vector plasmid (pCAGGS) or NSs expression plasmid (pCAGGS-NSs) **(C)**, and collected at the indicated time points post infection or transfection for WB analyses. Using the ImageJ software, protein band intensities of STAT1 and STAT2 from **(A,C)** were measured and then normalized to those of β-actin, as shown in **(B,D)**, respectively. Note that the relative band intensities at 0 h post infection or transfection were set to 1 as indicated by the dotted reference lines. **(E)** HEK293 cells transfected with the S-tagged STAT1 (STAT1-S) expression plasmid or empty vector were infected with SFTSV or mock infected. At 24 h p.i., cells were lysed for S-pulldown assays, followed by WB analysis with the indicated antibodies. **(F)** Cells were transfected with plasmids encoding the indicated viral proteins or the control vector. At 72 h posttransfection, cells were harvested and subjected to WB analysis using antibodies against the indicated proteins. **(G)** Relative band intensities of STAT1 from **(F)** were analyzed using ImageJ. Dotted reference line indicates the ordinate value 1. **(H)** HEK293 cells were mock infected or infected with SFTSV for the indicated times. Relative mRNA levels of the cellular proteins in the infected cells (normalized to mock-infected groups at the corresponding time points) were analyzed by real-time quantitative PCR. *B-cell CLL/lymphoma factor* (*BCL*), a virus-induced gene control. Dotted line indicates the ordinate value 1 for reference. Data are shown as means ± SD, *n* = 3.

To further characterize SFTSV infection-induced down-regulation of STAT1, host protein levels in mock- or SFTSV-infected cells were respectively monitored by time-course WB analyses. As shown in Figures 7A,B, the STAT1 protein abundance was indeed specifically reduced after 48 h with SFTSV infection. In contrast, neither the STAT1 in the mock infection group nor another transcription factor STAT2 exhibited such reduction (Figures 7A,B). Moreover, NSs expression alone by transient transfection did not noticeably decrease the STAT1 protein level (Figures 7C,D), consistently with the observations in the IFA. To further analyze the potential role of viral proteins in the STAT1 depletion, we next examined interaction of all the viral proteins (NSs, NP, GP, and RdRp) with STAT1 by S-pulldown assay with S-tagged STAT1 as the bait. As indicated in Figure 7E, only NSs but not the other viral proteins was coprecipitated with STAT1, further confirming NSs-STAT1 interaction and also excluding the ability of the other SFTSV proteins to interact with STAT1. Then, as expected, like NSs, the structural proteins exhibited no obvious influence on STAT1 abundance either (Figures 7F,G). Furthermore, to test whether the reduction of STAT1 was resulted from down-regulation of mRNA expression, mRNA levels of the host factors were then examined by real-time quantitative PCR. Interestingly, SFTSV infection resulted in moderate increase rather than reduction in *STAT1* and *STAT2* mRNA expression (Figure 7H), suggesting that STAT1 protein loss could not be attributed to mRNA level changes. Taken together, these results not only confirm SFTSV–infection-caused STAT1 protein depletion but also indicate that viral proteins themselves cannot or are insufficient to down-regulate STAT1 abundance and hence viral infection process or other infection product(s) may be involved in the STAT1 protein reduction.

### IFN-γ Pretreatment Confers Protection to Mice Against Lethal SFTSV Infection *in vivo*

**Figure 8:**
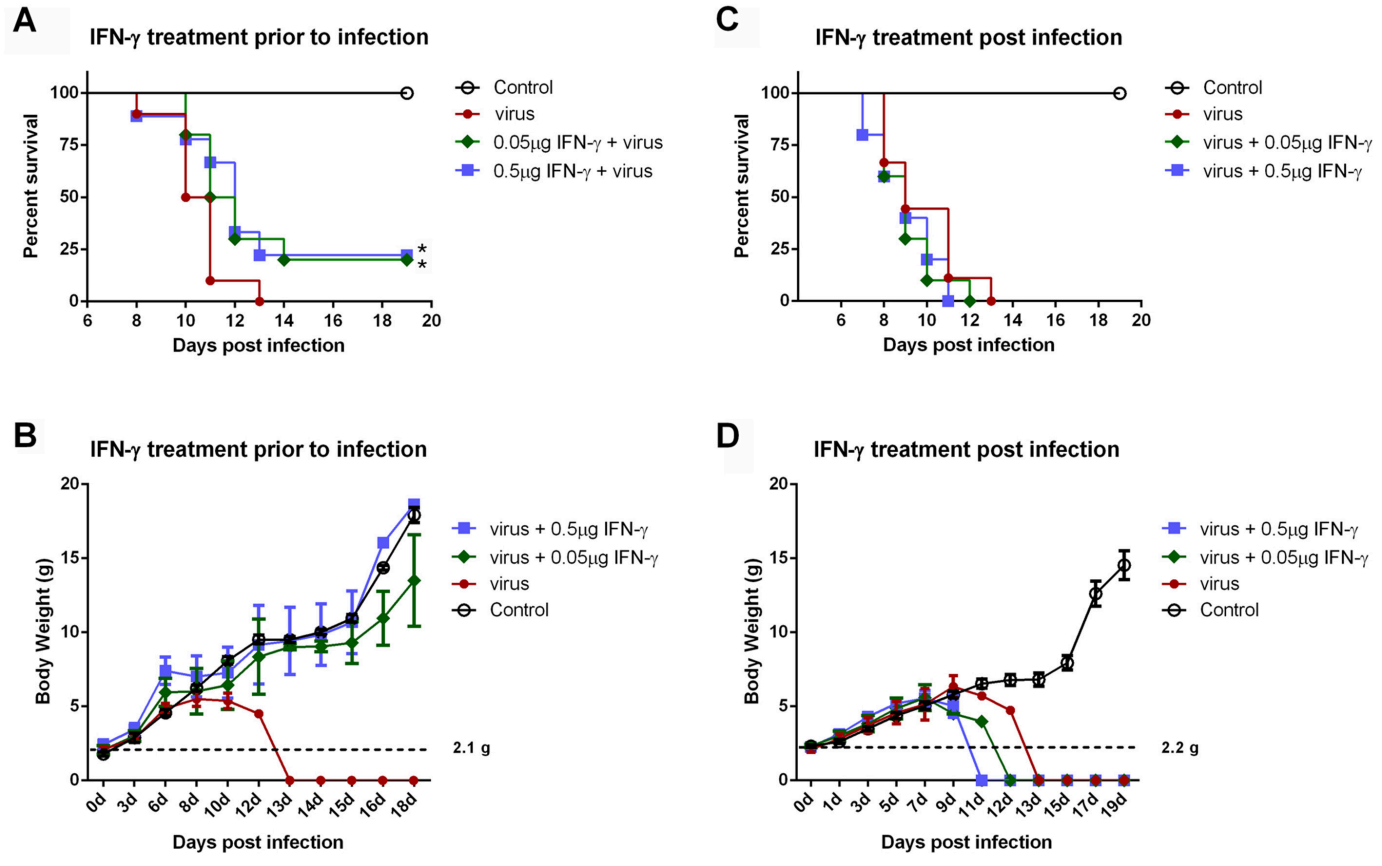
IFN-γ pretreatment confers protection to mice against lethal SFTSV infection *in vivo*. **(A,B)** Indicated doses of IFN-γ were administered to suckling mice (*n* ≥ 9/treatment) 24 h prior to infection of SFTSV (1.5×10^3^ TCID50). **(C,D)** Indicated doses of IFN-γ were administered 24 h following SFTSV inoculation (*n* ≥ 8/treatment). Control animals were injected with culture medium instead of virus. The survival rates and body weights of survived mice were monitored and recorded at the indicated times following virus challenge. Dotted line represents the average weight of the neonatal mice for virus challenge. Statistical conclusions obtained from Mantel-Cox log-rank and Gehan-Breslow-Wilcoxon tests were unanimous compared to the virus-infected group without IFN-γ treatment, *P < 0.05.

IFN-γ is an FDA-approved drug. As demonstrated above, IFN-γ exhibited antiviral activities against SFTSV infection at the cellular level; moreover, SFTSV seems to have developed the strategies to diminish the action of IFN-γ, which in turn may also reflect the potential role of IFN-γ signaling as an obstacle to SFTSV infection. Accordingly, assessment of the IFN-γ efficacy as a potential anti-SFTSV drug *in vivo* should be merited. Therefore, we next analyzed the ability of IFN-γ to protect suckling mice against lethal SFTSV challenge. Suckling mice were used because they can be efficiently infected by SFTSV and consequently exhibit significant morbidity and mortality, while little to no pathogenesis is observed in SFTSV-challenged adult mice (44, 45). Mouse IFN-γ (0.5 or 0.05 μg per animal) was administrated prior to or after SFTSV infection and the survival rates and body weights were monitored in the following days. IFN-γ treatment prior to SFTSV infection significantly reduced mortality, protecting ~25% of the animals from death, whereas all the untreated mice died off in 13 days with SFTSV challenge (Figure 8A). Moreover, IFN-γ pretreatment obviously prevented body weight loss and the body weights of survived mice were steadily increased (Figure 8B), confirming a certain anti-SFTSV activity of IFN-γ *in vivo*. Nevertheless, no protective effect on survival rates or body weights was observed in the mice treated with IFN-γ after 24 h with SFTSV infection, manifesting the resistance of SFTSV to IFN-γ antiviral action after the viral infection establishment, in line with the findings of our cell-based assays. Collectively, these data suggest that IFN-γ indeed has anti-SFTSV activity *in vivo* when used prophylactically, whereas the antiviral activity can be neutralized after SFTSV infection establishment due to the viral antagonism of IFN-γ-STAT1 signaling.

## Discussion

**Figure 9:**
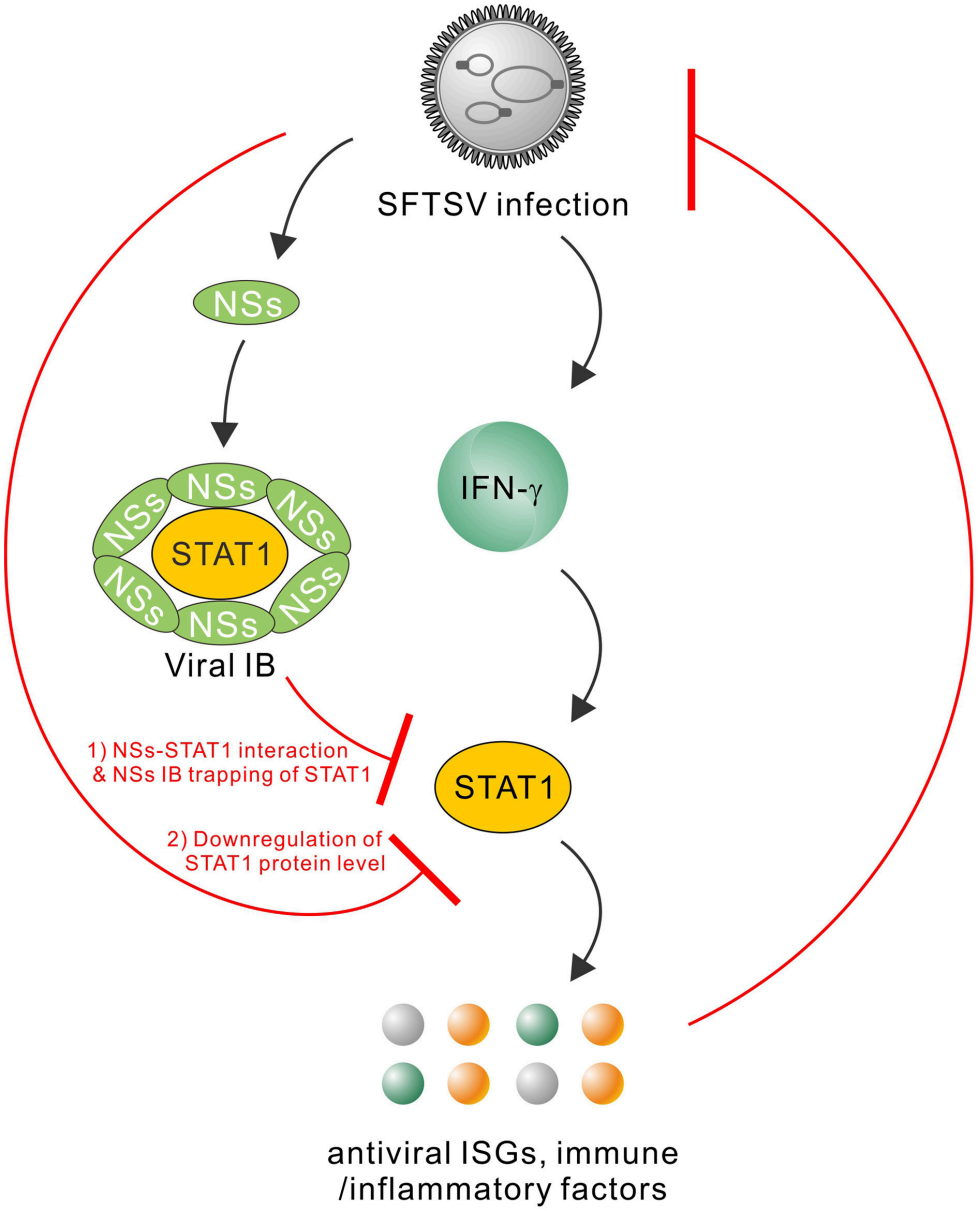
Model for the interplays between SFTSV infection and IFN-γ-STAT1 signaling. SFTSV infection leads to the substantial production of IFN-γ which can direct anti-SFTSV action through induction of antiviral ISGs and other immune or inflammatory factors by IFN-γ-STAT1 signaling. In turn, the host anti-SFTSV response mounted by IFN-γ can be counteracted by the virus through the following two mechanisms: (1) NSs IB sequestration of STAT1 by the NSs-STAT1 interaction and (2) virus infection-induced downregulation of STAT1 protein abundance. Together, these viral and cellular actions reflect a complex arm race between SFTSV and its host, shedding lights on the virus-host interactions and viral pathogenesis.

Type I and III IFNs have general antiviral activities, whilst it has been reported that numerous viruses including SFTSV have developed diverse strategies to circumvent the antiviral signaling by these IFNs (20, 46–49). In contrast, IFN-γ shows antiviral efficacy *in vivo* to only several viruses (24, 50–52) and meanwhile, there are fewer reports on viral antagonism of IFN-γ responses (52). Here, we are the first to explore the effect of type II IFN, IFN-γ, on SFTSV infection. Firstly, we detected the significant induction of IFN-γ expression in SFTS patient sera, indicating the remarkable host IFN-γ responses. Then, we demonstrated that IFN-γ indeed has the anti-SFTSV efficacy, especially when used before the viral infection establishment. There is currently no specific vaccine or antiviral against SFTSV, while the present study indicates some potential of IFN-γ as a prophylactic drug against the lethal viral infection. However, the action of IFN-γ is compromised or lost when used after SFTSV infection establishment. Subsequently, we showed that SFTSV has armed with a complex IFN-γ antagonism capacity. SFTSV can abate IFN-γ signaling through (1) the NSs-STAT1 interaction-mediated sequestration of STAT1 in viral IBs and (2) viral infection-induced down-regulation of STAT1 protein abundance. These findings complement our knowledge of the interactions between SFTSV and IFN system and present an interesting picture of the virus-host arm race (Figure 9), providing new insights into IFN-γ antiviral biology and SFTSV pathogenesis.

The present study demonstrates the anti-SFTSV efficacy of IFN-γ both *in vitro* and *in vivo*, while we consider that the mechanistic processes underlying the IFN-γ action should be miscellaneous, likely involving multiple direct and indirect effectors (19, 23, 53). IFN-γ can directly stimulate the expression of some potential antiviral ISGs by the STAT1 signaling (23, 50, 54, 55), which likely contributes to the anti-SFTSV effect of IFN-γ as a direct mechanism as suggested by the cell-based assays (Figure 2). It will be interesting to further determine which and how ISGs are involved therein. In addition to the induction of antiviral proteins, IFN-γ has notable immunostimulatory and immunomodulatory activities including enhancing phagocytosis and antigen presentation, activating NK cells, driving CD4+ Th1 T cell development, and assisting the innate and adaptive immune responses (19, 53). These additional effects of IFN-γ may be also implicated in its antiviral activities under physiological conditions of viral systematic infections.

Recently, Rhein et al. reported the antiviral activity of IFN-γ in mice against Ebola virus (EBOV) infection (50). In the report, IFN-γ conferred more than 50% protection to mice from lethal challenge of mouse-adapted EBOV (50). In comparison, although IFN-γ exhibits a solid anti-SFTSV activity, the protection ratios of IFN-γ pretreatment against lethal SFTSV infection are only approaching 25%. Firstly, it should be noted that in order to avoid any noticeable side effects to the experimental animals, the administrated dosages of IFN-γ in our experiments are relatively lower compared to previous studies (50). It may be merited to further explore lower lethal infection dosages of SFTSV, higher safe dosages of IFN-γ, or other administration approaches or animal infection models in the future. Furthermore, the antagonistic capacity of SFTSV against IFN-γ-STAT1 signaling as shown in the current study may also explain the compromised protection efficacy, even in the pretreatment experiments. Additionally, although there are no statistical differences, IFN-γ administration at 24 h post SFTSV infection seemed to have a slight negative impact on the animal survival instead of protective effects (Figures 8C,D), probably reflecting the other side of IFN-γ as a pro-inflammatory factor which may exacerbate the inflammatory pathogenesis in some viral infections (53, 56, 57). However, against EBOV infection, IFN-γ treatment post viral challenge, by contrast, exhibited more efficacious protection than prophylactic administration. It likely implies some difference of the inflammatory pathogenesis of the two hemorrhagic fever viruses. The potential roles of IFN-γ in SFTSV pathogenesis (including the inflammatory pathogenesis) need further detailed investigation. Despite all this, in the future clinical therapeutic research on SFTS, IFN-γ still can be an alternative antiviral when elaborately used with appropriate administration approaches, alone or in combination with other agents.

Previously, STAT2 was identified as the main target of IFN-α and IFN-β signaling suppression by SFTSV and meanwhile STAT1 was also considered as a possible target for viral inhibition of the type I IFN signaling due to an IB hijacking (13); here, we further demonstrate that SFTSV can also interfere with IFN-γ anti-SFTSV signaling by targeting STAT1 in proteomic perspective. STAT1, but not STAT2, is shared by the whole IFN family for the signaling cascades (54). Thus, in addition to giving the mechanistic explanation for SFTSV counteraction against IFN-γ, the present findings (particularly the virus infection-caused downregulation of STAT1 protein abundance) complement the knowledge of the viral antagonism strategies against type I and III IFNs as well. Furthermore, STAT1 is involved in the signaling by multiple interleukins and growth factors besides IFNs (54). It will be interesting to analyze whether other cytokine signaling pathways and hence the corresponding biological processes involving STAT1 are disturbed by SFTSV.

Aside from the NSs-STAT1 interaction and NSs IB trapping of STAT1, SFTSV infection specifically induces the downregulation of STAT1 at the protein level (Figures 6, 7). We previously observed an NSs-STAT1 colocalization but did not detect any STAT1 enrichment in the NSs immunoprecipitates of virus-infected cells (thus failing to unravel the direct targeting of STAT1 by NSs) (13), which can be reasonably explained here by the STAT1 loss in the context of viral infection. Interestingly, NSs expression itself could not reduce STAT1 abundance, although NSs is the sole viral protein that can target STAT1 by protein interaction. Furthermore, expression of the other viral proteins (NP, GP, or RdRp) could not affect STAT1 protein level either, suggesting that viral proteins themselves cannot or are insufficient to induce STAT1 reduction and viral infection process or some other specific events of virus life cycle should be implicated therein. SFTSV-infection-mediated STAT1 protein reduction is reminiscent of the specific degradation of STAT1 (but not STAT2) induced by parainfluenza virus 5 (58) and mumps virus (59) through proteasomal pathway. However, the proteasome-mediated STAT1 degradation was rapid and could be observed in the first several hours after viral infections (~4–10 h p.i.) (58, 59), while SFTSV infection resulted in STAT1 abundance decrease with rather different kinetics (Figure 7A) and the protein reduction was noticed at 24–48 h p.i. and later, indicating that the other cellular processes such as the lysosomal degradation pathways may be involved. In addition to protein degradation pathways, transcriptional or post-transcriptional processes of host cells also are often affected by viral infections, which can lead to protein level changes as well. In the present study, we showed that the STAT1 downregulation could not be attributed to transcriptional changes, as SFTSV infection led to a moderate increase (but not decrease) of STAT1 mRNA level (Figure 7C). It is not surprised as to STAT1 mRNA upmodulation because STAT1 itself is an ISG which can be most often induced by virus infections unavoidably. Despite that, potential post-transcriptional modulation (such as changes of mRNA methlytion patterns) by virus infection that may also contribute to some host protein downregulation still cannot be excluded either. Thus, to further unravel the mechanism(s) of STAT1 depletion, comprehensive analyses on the aforementioned cellular processes potentially influenced by SFTSV infection will be merited in future work. To our knowledge, SFTSV is the first member of the huge *Bunyavirales* order shown here to be able to down-regulate STAT1 protein abundance. The present findings as well as further exploration of the involved viral and cellular processes could offer new insights into bunyavirus manipulation of host factors at the protein level.

Following the identification of SFTSV, another pathogenic phlebovirus genetically closely related to SFTSV, named Heartland virus (HRTV), was recognized in the United States (60). SFTSV and HRTV, together with other newly isolated phleboviruses have constituted the SFTSV/HRTV-related virus group (61). These emerging phleboviruses are posing serious threats to the worldwide public health. It will be merited to investigate whether IFN-γ has antiviral activity against the other SFTSV-related viruses and whether these viruses have evolved the capacity for IFN-γ antagonism like SFTSV.

In summary, the current study demonstrates that the type II IFN, IFN-γ, which can be substantially induced during SFTSV infection has anti-SFTSV efficacy both *in vitro* and *in vivo*; in turn, the antiviral activity is counteracted by SFTSV through NSs-STAT1 interaction-mediated sequestration of STAT1 in viral IBs and virus infection-induced downregulation of STAT1 protein abundance. These viral and cellular activities collectively reflect a picture of virus-host arm race regarding to IFN-γ antiviral immunity and viral targeted counteraction. The new insights toward understanding virus-host mutual manipulations as gained in this work provide critical clues to the viral pathogenesis and antiviral intervention studies.

## Ethics Statement

The clinical serum analysis was approved by both the ethics committees of Wuhan Institute of Virology, Chinese Academy of Sciences and the Union Hospital of Tongji Medical College, Huazhong University of Science and Technology. Written informed consent was obtained from the human subjects involved prior to the serum collection. The animal experiments were approved by the Institutional Animal Ethical Committee of Wuhan Institute of Virology, Chinese Academy of Sciences and carried out under the guidelines of the Care and Use of Laboratory Animals (the Ministry of Science and Technology, China).

## Author Contributions

HW supervised the research. Y-JN conceived the study and design experiments. QM, KF, Y-QM, DH, and Y-JN performed the experiments. Y-JN, QM, KF, Y-QM, FD, ZH, and HW analyzed the data. ML, CP, XZ, FD, ZH, and HW provided materials and contributed to completion of the study. Y-JN wrote the manuscript. All authors reviewed the results, read and approved the final manuscript version.

### Conflict of Interest Statement

The authors declare that the research was conducted in the absence of any commercial or financial relationships that could be construed as a potential conflict of interest.
